# Hierarchical Modelling of Haplotype Effects on a Phylogeny

**DOI:** 10.3389/fgene.2020.531218

**Published:** 2021-01-15

**Authors:** Maria Lie Selle, Ingelin Steinsland, Finn Lindgren, Vladimir Brajkovic, Vlatka Cubric-Curik, Gregor Gorjanc

**Affiliations:** ^1^Department of Mathematical Sciences, Norwegian University of Science and Technology (NTNU), Trondheim, Norway; ^2^School of Mathematics, University of Edinburgh, Edinburgh, United Kingdom; ^3^Department of Animal Science, Faculty of Agriculture, University of Zagreb, Zagreb, Croatia; ^4^The Roslin Institute and Royal (Dick) School of Veterinary Studies, University of Edinburgh, Edinburgh, United Kingdom

**Keywords:** genealogy, haplotype, DAG, autoregression, INLA, Bayesian

## Abstract

We introduce a hierarchical model to estimate haplotype effects based on phylogenetic relationships between haplotypes and their association with observed phenotypes. In a population there are many, but not all possible, distinct haplotypes and few observations per haplotype. Further, haplotype frequencies tend to vary substantially. Such data structure challenge estimation of haplotype effects. However, haplotypes often differ only due to few mutations, and leveraging similarities can improve the estimation of effects. We build on extensive literature and develop an autoregressive model of order one that models haplotype effects by leveraging phylogenetic relationships described with a directed acyclic graph. The phylogenetic relationships can be either in a form of a tree or a network, and we refer to the model as the haplotype network model. The model can be included as a component in a phenotype model to estimate associations between haplotypes and phenotypes. Our key contribution is that we obtain a sparse model, and by using hierarchical autoregression, the flow of information between similar haplotypes is estimated from the data. A simulation study shows that the hierarchical model can improve estimates of haplotype effects compared to an independent haplotype model, especially with few observations for a specific haplotype. We also compared it to a mutation model and observed comparable performance, though the haplotype model has the potential to capture background specific effects. We demonstrate the model with a study of mitochondrial haplotype effects on milk yield in cattle. We provide R code to fit the model with the INLA package.

## 1. Introduction

This paper develops a hierarchical model to estimate haplotype effects based on phylogenetic relationships between haplotypes and their association with observed phenotypes. With current technology we can readily obtain genome-wide information about an individual, either through single-nucleotide polymorphism array genotyping or sequencing platforms. Since the genome-wide information has become abundant, modelling this data has become the standard in animal and plant breeding as well as human genetics. The application of this modelling has been shown to improve genetic gains in breeding (Meuwissen et al., 2001; Ibanez-Escriche and Simianer, 2016; Hickey et al., 2017), and has potential for personalised prediction in human genetics and medicine (de los Campos et al., 2018; Lello et al., 2018; Maier et al., 2018; Begum, 2019).

Geneticists aim to infer which mutations are causing variation in phenotypes and what are their effects. This aim is nowadays approached with genome-wide association studies of regressing observed phenotypes on mutation genotypes (Morris and Cardon, 2019). However, mutations arise on specific haplotypes passed between generations, which limits accurate estimation due to low frequency of mutations, correlation with other mutations and limited ability to observe all mutations with a used genomic platform (e.g., see Gibson, 2018; Simons et al., 2018; Uricchio, 2019). Further, most mutations do not affect phenotypes, while some mutations have background (haplotype) specific effects (e.g., Chandler et al., 2017; Steyn et al., 2019; Wojcik et al., 2019).

Instead of focusing on mutation effects we here focus on haplotype effects and their differences to estimate the effect of mutations on specific haplotypes. There is extensive literature on estimating haplotype effects (Balding, 2006; Thompson, 2013; Morris and Cardon, 2019). One issue with estimating haplotype effects is that there is usually an uneven distribution of haplotypes in a population (Ewens, 1972, 2004; Walsh and Lynch, 2018), and estimating the effects of rare haplotypes is equally challenging as estimating the effect of rare mutations. However, the described genetic processes in the previous paragraph create a “network” of haplotypes (sometimes referred to as *genealogy* or *phylogeny*), which suggests that effects of similar haplotypes are similar. This observation inspired (Templeton et al., 1987) to cluster phylogenetically similar haplotypes. Others have used similar approaches to account or leverage haplotype similarities (Balding, 2006; Thompson, 2013; Morris and Cardon, 2019).

We here approach the problem of estimating haplotype effects by leveraging phylogenetic relationships between haplotypes described with a directed acyclic graph (DAG) (Koller and Friedman, 2009) and developing a hierarchical model of haplotype effects on this graph. We were inspired by recent advances in building phylogenies on large data sets (Kelleher et al., 2019), and aimed to develop a hierarchical model that could scale to a large number of haplotypes. Our work extends the phylogenetic mixed modelling of the whole genome (Lynch, 1991; Pagel, 1999; Housworth et al., 2004; Hadfield and Nakagawa, 2010) to a specific region. This region specific modelling could be applied either across species (macroevolution) or within a species (microevolution).

A potentially important modelling aspect with respect to across and within species modelling is that the phylogenetic mixed model assumes Brownian motion for evolution of phenotypes along a phylogeny (Felsenstein, 1988; Huey et al., 2019). Brownian motion is a continuous random-walk process with variance that grows over time (is non-stationary) (Gardiner, 2009; Blomberg et al., 2019), which makes it a plausible model of evolution due to mutation and drift. There are alternatives to Brownian motion, in particular the Ornstein-Uhlenbeck process that can accommodate various forms of selection (Lande, 1976; Hansen and Martins, 1996; Martins and Hansen, 1997; Paradis, 2014). The Ornstein-Uhlenbeck process is also a continuous random-walk, but with an additional parameter that reverts the process to the mean (is a stationary process; e.g., Gardiner, 2009; Blomberg et al., 2019). Both of these models imply Gaussian distributions for the initial state and increments. The differences between the two processes might be important in the context of modelling haplotypes that likely manifest less variation than whole genomes, particularly when considering haplotypes within a species or even a specific population.

The aim of this paper is to develop a hierarchical model for haplotype effects by leveraging phylogenetic relationships between haplotypes. We assume that such relationships are described with a DAG encoded network and therefore call the model the haplotype network model. Since haplotypes differ due to a small number of mutations and very few mutations have an effect we expect that phylogenetically similar haplotypes will have similar effects. Furthermore, the small discrete number of mutation differences suggest discrete-time analogues of Brownian and Ornstein-Uhlenbeck processes. Therefore, we have modelled the effect of a mutated haplotype given its parental haplotype with a stationary autoregressive model of order one following the phylogenetic structure encoded with a DAG. The results show that the haplotype network model improves the estimation of haplotype effects compared to an independent haplotype model due to sharing of information. The results also show that it is comparable to a mutation model, but has the potential to capture background specific effects.

## 2. Materials and Methods

We present the haplotype network model and show how to use it as a component in a phenotype model. We also describe simulations, a case study of modelling mitochondrial effects on milk yield in cattle, and the chosen method to perform inference and model evaluation.

### 2.1. The Haplotype Network Model

We present the haplotype network model, which is a hierarchical model for haplotype effects based on phylogenetic relationships between haplotypes encoded with a DAG. The phylogenetic relationships can be either in a form of a tree or a more general network. We also present two generalisations of the model—first due to multiple parental haplotypes and second due to genetic recombination. By multiple parental haplotypes we mean the situation where two different haplotypes in a phylogeny mutate into the same haplotype.

We assume throughout that the phylogeny between haplotypes is known and that it can be encoded with a DAG. The haplotype network model can in principle deal with different types of mutations, but for simplicity we focus only on biallelic mutations with the code 0 used for the ancestral/reference allele (commonly at a higher frequency in a population), and the code 1 used for the alternative allele that arose due to a mutation.

#### 2.1.1. Motivating Example

**Figure 1:**
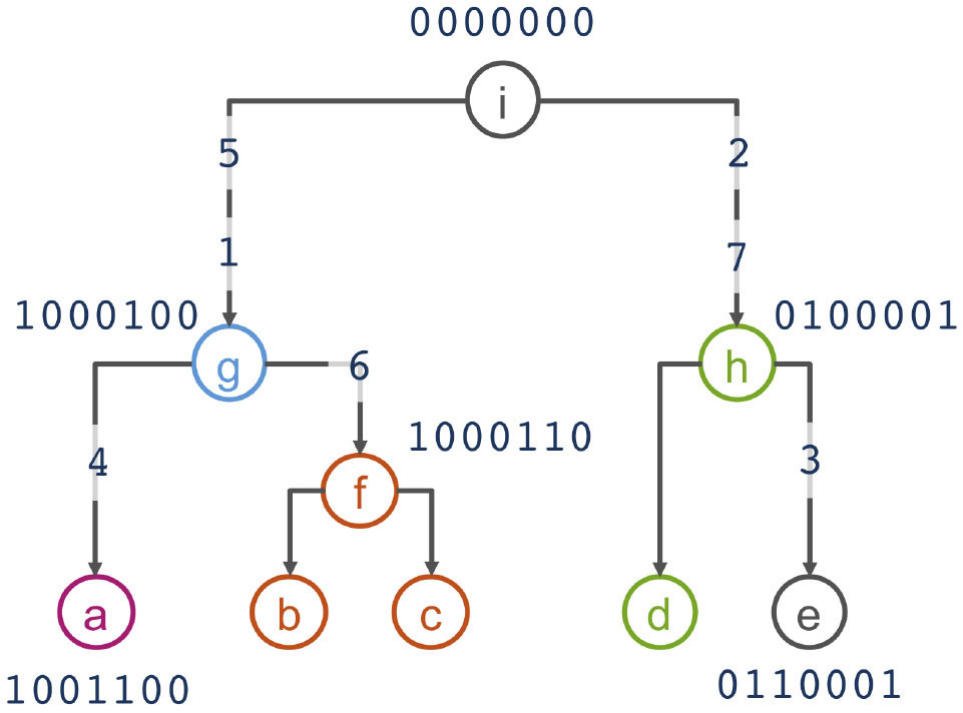
Phylogenetic relationship of haplotypes in Table 1.

To motivate the haplotype network model, we use the example from Kelleher et al. (2019) that presents 5 haplotypes spanning 7 biallelic polymorphic sites (Table 1). Note that the 5 haplotypes are just a sample of the 2^7^ = 128 possible haplotypes over the 7 sites. An example of a phylogeny for the haplotypes is shown in Figure 1, where haplotypes are denoted as nodes (we also show their allele sequence), relationships between haplotypes are denoted as edges, and mutated sites are denoted with a number on edges. For example, the ancestral haplotype *i* has allele sequence 0000000, and the haplotype *g* with sequence 1000100 differs from the ancestral haplotype due to mutations at the sites 5 and 1.

**Table 1:** Example of 5 haplotypes spanning 7 mutations from Kelleher et al. (2019).

		**Site**						
		**1**	**2**	**3**	**4**	**5**	**6**	**7**
Haplotype	a	1	0	0	1	1	0	0
	b	1	0	0	0	1	1	0
	c	1	0	0	0	1	1	0
	d	0	1	0	0	0	0	1
	e	0	1	1	0	0	0	1

*The ancestral (reference) alleles are coded as 0 and alternative alleles are coded as 1*.

Assuming that similar haplotypes have similar effects, we model dependency between parent-progeny pairs of haplotypes with an autoregressive Gaussian process of order one. For haplotypes in Table 1 and Figure 1 this model implies the following set of conditional dependencies:

$$\begin{array}{l} \begin{array}{ccc} h_{i} & \sim & N(0,\sigma_{h_{m}}^{2})\\ h_{g^{\prime }}|h_{i} & \sim & N(\rho h_{i},\sigma_{h_{c}}^{2})\\ h_{g}|h_{g^{\prime }} & \sim & N(\rho h_{g^{\prime }},\sigma_{h_{c}}^{2})\\ h_{a}|h_{g} & \sim & N(\rho h_{g},\sigma_{h_{c}}^{2})\\ h_{f},h_{b},h_{c},|h_{g} & \sim & N(\rho h_{g},\sigma_{h_{c}}^{2})\\ h_{h^{\prime }}|h_{i} & \sim & N(\rho h_{i},\sigma_{h_{c}}^{2})\\ h_{h},h_{d}|h_{h^{\prime }} & \sim & N(\rho h_{h^{\prime }},\sigma_{h_{c}}^{2})\\ h_{e}|h_{h} & \sim & N(\rho h_{h},\sigma_{h_{c}}^{2}) \end{array} \end{array}$$

where *h*_*i*_, *h*_*g*_, …, *h*_*e*_ indicate the effect of haplotypes *i, g*, …, *e*, and $h_{{*}^{\prime }}$ indicates the effect of haplotypes that occur between haplotypes separated by multiple mutations, for example, *g*′ is the additional haplotype between the haplotypes *i* and *g* due to two mutations between *i* and *g*; we describe the other model parameters $\left(\rho ,\sigma_{h_{m}}^{2},\sigma_{h_{c}}^{2}\right)$ in the following.

#### 2.1.2. The Model

Assume a known general phylogenetic network of haplotypes described with a DAG with haplotype effects as nodes and relationships between the haplotype effects as edges as in Figure 1, and let repeated identical haplotypes be handled as the same haplotype. We model the effect of a chosen “starting” (this could be either a central, ancestral, most common or some other choice) haplotype 1 with mean-zero and marginal variance $\sigma_{h_{m}}^{2}$:

(1) $$\begin{array}{l} h_{1}\sim N\left(0,\sigma_{h_{m}}^{2}\right), \end{array}$$

and any other haplotype *j* in the phylogenetic network as a function of its one-mutation-removed parental haplotype *p*(*j*) assuming the autoregressive Gaussian process of order one with the autocorrelation between haplotype effects of ρ (|ρ| < 1 to ensure stationarity) and conditional variance of $\sigma_{h_{c}}^{2}$ as:

(2) $$\begin{array}{l} h_{j}|h_{p\left(j\right)}\sim N\left(\rho h_{p\left(j\right)},\sigma_{h_{c}}^{2}\right). \end{array}$$

We consider the autoregressive Gaussian process of order one that is stationary both in mean and variance, which is achieved by setting the marginal variance to $\sigma_{h_{m}}^{2}=\sigma_{h_{c}}^{2}/\left(1-\rho^{2}\right)$, so $\sigma_{h_{c}}^{2}=\sigma_{h_{m}}^{2}\left(1-\rho^{2}\right)$. The variance parameter is capturing scale (spread) of haplotype effects and the autocorrelation parameter is capturing dependency between haplotype effects. This is the standard autoregressive model of order one used in time-series analysis (e.g., Rue and Held, 2005). The difference here is that we are applying the model onto a phylogenetic network described with a tree or more generally with a DAG (Basseville et al., 1992; Wu et al., 2020).

The set of distributions in Equation (1) and Equation (2) give a system of equations for all *n* haplotype effects $h={\left(h_{1},...,h_{n}\right)}^{T}$:

(3) $$\begin{array}{l} h=\text{T}\left(\rho \right)\varepsilon , \end{array}$$

(4) $$\begin{array}{l} \text{T}{\left(\rho \right)}^{-1}h=\varepsilon , \end{array}$$

where the matrices **T**(ρ) and **T**(ρ)^−1^ of dimension *n* × *n* respectively represent marginal and conditional phylogenetic regression between haplotype effects ***h*** and the vector ε represents haplotype effect deviations, $\varepsilon \sim N\left(0,\text{D}\left(\rho \right)\sigma_{h_{c}}^{2}\right)$. The expression **T**(ρ) indicates that the matrix **T** depends on the value of ρ. Since haplotype effect deviations are independent, the matrix **D**(ρ) is diagonal and has value 1/(1 − ρ^2^) for the “starting” haplotype and 1 for the other haplotypes. Following the assumed autoregressive process of order one (2), the non-zero elements of **T**(ρ)^−1^ are 1 along the diagonal and −ρ between a haplotype effect (row index) and its parental haplotype effect (column index). This simple sparse lower-triangular structure of the matrix **T**(ρ)^−1^ arises from the Markov properties of the autoregressive process (Rue and Held, 2005).

From Equation (3), the covariance between haplotype effects is:

(5) $$\begin{array}{l} \text{Var}\left(h\right)=\text{Var}\left(\text{T}\left(\rho \right)\varepsilon \right), \end{array}$$

(6) $$\begin{array}{l} =\text{T}\left(\rho \right)\text{Var}\left(\varepsilon \right)\text{T}{\left(\rho \right)}^{T}=\text{T}\left(\rho \right)\text{D}\left(\rho \right)\text{T}{\left(\rho \right)}^{T}\sigma_{h_{c}}^{2} \end{array}$$

(7) $$\begin{array}{l} =\text{H}\left(\rho \right)\sigma_{h_{c}}^{2}={\text{V}}_{h}\left(\rho ,\sigma_{h_{c}}^{2}\right), \end{array}$$

The covariance expression in Equation (5) shows that haplotype covariances ${\text{V}}_{h}\left(\rho ,\sigma_{h_{c}}^{2}\right)$ depend on the autocorrelation and variance parameters, while the covariance coefficients **H**(ρ) depend only on the autocorrelation parameter. Note that the parameters ρ and $\sigma_{h_{c}}^{2}$ are correlated by definition $\sigma_{h_{c}}^{2}=\sigma_{h_{m}}^{2}\left(1-\rho^{2}\right)$. When ρ = 0 there is no covariance between haplotype effects due to phylogenetic relationships, which suggests a model where haplotype effects are identically and independently distributed, $h\sim N\left(0,\text{I}\sigma_{h_{m}}^{2}\right)$. When ρ ≠ 0 effects of phylogenetically related haplotypes covary due to shared mutations.

For completeness, the joint density of all *n* haplotype effects ***h*** is multivariate Gaussian:

(8) $$\begin{array}{ll} h|\rho ,\sigma_{h_{c}}^{2} & \sim N\left(0,{\text{V}}_{h}\left(\rho ,\sigma_{h_{c}}^{2}\right)\right), \end{array}$$

with the probability density function:

(9) $$\begin{array}{l} p\left(h|\rho ,\sigma_{h_{c}}^{2}\right)={\left(\frac{1}{\sqrt{2\pi }}\right)}^{n}\sigma_{h_{c}}^{-n}{\left(1-\rho^{2}\right)}^{1/2}\\ \;\exp\left(-\frac{1}{2\sigma_{h_{c}}^{2}}h^{T}{\text{H}\left(\rho \right)}^{-1}h\right). \end{array}$$

The expression in Equation (9) involves inverse of the covariance coefficient (precision) matrix **H**(ρ)^−1^, which we can obtain without computationally expensive inverse of the **H**(ρ) (5). Following the definition in Equation (5), inverting both sides and using the described structure of **T**(ρ)^−1^ available from the DAG and **D**(ρ), we can efficiently get this inverse by:

(10) $$\begin{array}{l} {\text{H}\left(\rho \right)}^{-1}=\frac{1}{\sigma_{h_{c}}^{2}}{{\text{T}\left(\rho \right)}^{-1}}^{T}{\text{D}\left(\rho \right)}^{-1}{\text{T}\left(\rho \right)}^{-1}. \end{array}$$

Inspection of the structure of Equation (10) shows that this is a very sparse matrix with a structure. We can compute the non-zero elements of $\sigma_{h_{c}}^{2}\text{H}{\left(\rho \right)}^{-1}$ directly with the following simple algorithm where we loop over all haplotypes:

  **if** the haplotype is the “starting” haplotype **then**
     add 1 − ρ^2^ to the diagonal element
  **else**
     add 1 to the diagonal element
  **end if**
  **if** the haplotype has a parental haplotype **then**
     set off-diagonal element between the haplotype and its parental haplotype to −ρ
     add ρ^2^ to the diagonal element of the parental haplotype
  **end if**

To fully specify the model for ***h*** in Equation (8), prior distributions must be assigned to the autocorrelation parameter ρ and the marginal variance $\sigma_{h_{m}}^{2}$ or the conditional variance $\sigma_{h_{c}}^{2}$. Because most mutations do not have an effect we can expect that most parent-progeny pairs of haplotypes will have similar effects, which suggests that the autocorrelation parameter will be close to 1. This knowledge can be incorporated in the prior distribution for ρ. For the variance parameters there may be some prior knowledge about the size of haplotype effects relative to other effects, which can also be taken into account when choosing the prior distribution. We will specify prior distributions for these parameters in later sections.

#### 2.1.3. Multiple Parental Haplotypes

Sometimes phylogenetic inference cannot resolve bifurcating trees with dichotomies (one parental haplotype and two progeny haplotypes) and outputs a multifurcating tree with polytomies (one parental haplotype and multiple progeny haplotypes) or even just a network [multiple parent haplotypes and multiple progeny haplotypes (e.g., Schliep et al., 2017; Uyeda et al., 2018)]. The multiple progeny case works out of the box with the initial model, and we will here present an extension of the model presented in section 2.1.2, that can accommodate the multiple parent haplotypes and multiple progeny haplotypes case where the trees or networks can be described with a DAG.

We assume that the effects of all ancestral haplotypes, the haplotypes at the top of the network, are independent and come from the same Gaussian distribution $N\left(0,\text{I}\sigma_{h_{m}}^{2}\right)$. We further assume conditional independence between a haplotype and all previous haplotypes in the network given the parents of that haplotype. In the model where each haplotype had only a single parent haplotype it was assumed that the haplotype effect was ρ times the parental haplotype effect plus some Gaussian noise. When a haplotype has multiple parents, we now assume that the effect is the average over each of these processes from each parental haplotype.

We illustrate this with a small example which implies the model construction used. Let haplotype segment *d* have parental haplotypes segments *a*, *b*, and *c*. We denote the contribution from each of these parental segments *h*_*d*_*a*__, *h*_*d*_*b*__, *h*_*d*_*c*__, and assume:

$$\begin{array}{l} h_{d_{a}}=\rho h_{a}+\varepsilon_{d_{a}}\\ h_{d_{b}}=\rho h_{b}+\varepsilon_{d_{b}}\\ h_{d_{c}}=\rho h_{c}+\varepsilon_{d_{c}} \end{array}$$

where ${\left(\varepsilon_{d_{a}},\varepsilon_{d_{b}},\varepsilon_{d_{c}}\right)}^{T}\sim N\left(0,\text{I}\sigma_{h_{c}}^{2}\right)$. Further, we assume that the resulting effect of haplotype *h*_*d*_ is the average over all parent processes:

$$\begin{array}{l} h_{d}=\frac{\rho }{3}\left(h_{a}+h_{b}+h_{c}\right)+\frac{1}{3}\left(\varepsilon_{d_{a}}+\varepsilon_{d_{b}}+\varepsilon_{d_{c}}\right). \end{array}$$

The distribution of *h*_*d*_ conditional on *h*_*a*_, *h*_*b*_, and *h*_*c*_ becomes:

$$\begin{array}{l} h_{d}|h_{d_{a}},h_{d_{b}},h_{d_{c}}\sim N\left(\frac{\rho }{3}\left(h_{a}+h_{b}+h_{c}\right),\frac{\sigma_{h_{c}}^{2}}{3}\right). \end{array}$$

In general this means that $h_{i}|h_{1},...,h_{k}\sim N\left(\frac{\rho }{k}\overset{k}{\underset{j=1}{\sum }}h_{j},\frac{\sigma_{h_{c}}^{2}}{k}\right)$, for haplotype *i* with parental haplotypes 1, ..., *k*. This model construction corresponds to a model where one first takes every path down through the DAG and assigns separate stationary autoregressive processes of order one to each such path, and then assume conditionally independent but identical autoregressive processes of order one, that is, the processes have the same parameters.

Multiple parental haplotypes change the structure of the **T**(ρ)^−1^ matrix to having −ρ/*k*_*i*_ value between a haplotype effect (row index) and its parental haplotype effect (column index) and **D**(ρ)^−1^ matrix diagonals for “non-starting” haplotypes to *k*_*i*_, where *k*_*i*_ is the number of parental haplotypes of the haplotype *i*. The algorithm to setup the $\sigma_{h_{c}}^{2}\text{H}{\left(\rho \right)}^{-1}$ matrix is then (looping over all haplotypes)

  **if** the haplotype is the “starting” haplotype **then**
     add to the diagonal element 1 − ρ^2^
  **else**
     add *k*_*i*_ to the diagonal element
  **end if**
  **if** the haplotype has a parental haplotype **then**
     set off-diagonal element between the haplotype and its parental haplotype to −ρ
     set off-diagonal elements between all parental haplotypes that share that progeny haplotype to $\rho^{2}/k_{i}$
     add $\rho^{2}/k_{i}$ to the diagonal element of the parental haplotype(s)
  **end if**

The model presented in this section is only one of many possible choices for a model accommodating multiple parental haplotypes. There are other options that could model such graph structures, for example by modelling it as a mixture distribution with variable probabilities between parental haplotypes.

#### 2.1.4. Expanding to Multiple Regions Due to Recombination

Haplotype phylogeny can differ along genome regions due to recombination—the process of swapping genome regions between haplotypes during meiosis. We accommodate this in the haplotype network model by considering each haplotype region separately, but still within the framework of the same model. This means that the effect of haplotype *h*_*i*_ is modelled as the sum of effects for all haplotype regions. Consider haplotypes spanning three regions. The effect of haplotype *i*, is then assumed to be the sum of the effects of haplotype segments in each of the three regions:

$$\begin{array}{l} h_{i}=h_{1,i}+h_{2,i}+h_{3,i}. \end{array}$$

We assume the haplotype network model for each haplotype region, but with joint hyper-parameters $\left(\rho ,\sigma_{h_{c}}^{2}\right)$. Let ***h*** = (*h*_1,1_, …, *h*_1,*n*_1__, *h*_2,1_, …, *h*_*m*,*n*_*m*__) be the effect of all haplotypes in all regions, where *m* is the number of regions and *n* is the number of haplotypes in each region. The joint probability density for the haplotype effects ***h*** is then:

$$\begin{array}{l} p\left(h|\rho ,\sigma_{h_{c}}^{2}\right)={\left(\frac{1}{\sqrt{2\pi }}\right)}^{n_{1}+\ldots +n_{m}}\sigma_{h_{c}}^{-\left(n_{1}+\ldots +n_{m}\right)}{\left(1-\rho^{2}\right)}^{m/2}\\ \;\exp\left(-\frac{1}{2\sigma_{h_{c}}^{2}}h^{T}{\text{H}\left(\rho \right)}^{-1}h\right), \end{array}$$

with:

(11) $$H{\left(\rho \right)}^{-1}=\left(\begin{array}{ccc} H{\left(\rho \right)}_{1}^{-1} & \; & \;\\ \; & \ddots & \;\\ \; & \; & H{\left(\rho \right)}_{m}^{-1} \end{array}\right).$$

Although recombination is common, we have focused on the special case of no recombination in this study, where the haplotypes are connected in one phylogeny, as presented in section 2.1.2. We address recombination in discussion.

### 2.2. Phenotype Model With Haplotype Effects

We now show how the haplotype effects can be included in a model for phenotypic observations. We also present a phenotype model that includes independent haplotype effects or mutation effects rather than the haplotypes.

Let ***y***_*p*×1_ be phenotype observations of *p* individuals and let ***h***_*n*×1_ be the effect of *n* haplotypes obtained from phasing genotypic data of the individuals. We assume the following model (Gaussian likelihood) for the centred and scaled phenotypic observations:

(12) $$\begin{array}{l} y_{p\times 1}={\text{X}}_{p\times r}\beta_{r\times 1}+f_{p\times 1}^{1}+...+f_{p\times 1}^{s}+Z_{p\times n}h_{n\times 1}+e_{p\times 1}, \end{array}$$

where β~N(0,I1000) is a vector of *r* fixed effects with covariate matrix **X**, $f\sim N\left(0,\text{I}\sigma_{f}^{2}\right)$ are random effects, ***h*** are the haplotype effects with incidence matrix **Z** that maps haplotypes to individuals, and the residual effect is $e\sim N\left(0,\text{I}\sigma_{e}^{2}\right)$. In the case of diploid individuals there will be two entries in every row of **Z**, and a single entry for haploid individuals or male sex chromosome or mitogenome.

We have assumed three different models for the haplotype effects ***h***. The first is a base model with independent haplotype effects (IH model), where $h\sim N\left(0,\text{I}\sigma_{h_{m}}^{2}\right)$. The second is the haplotype network model presented in section 2.1.2 (HN model), where $h\sim N\left(0,{\text{V}}_{h}\left(\rho ,\sigma_{h_{c}}^{2}\right)\right)$. The third is an alternative way of estimating haplotype effects via a linear combination of mutation effects (mutation model). Assume ***h*** = **U*v*** with $\nu \sim N\left(0,\text{I}\sigma_{v}^{2}\right)$ being mutation effects and **U** is the matrix containing the haplotype allele sequence with reference alleles coded as 0 and alternative alleles coded as 1. The effects described so far consist of the latent field of a Bayesian hierarchical model, and are assigned Gaussian prior distributions.

The models do not have a common intercept because a common intercept and the mean level in the haplotype effects are not identifiable when ρ approaches 1. Instead the mean level in the observations is captured by the haplotype effects, for computational reasons. A sum-to-zero constraint can be specified for the haplotype network part of the model if a common intercept is required, though this changes the model interpretation if ρ is close to 1. This problem is not specific to this model, but occurs for all autoregressive models when they are used as part of a structured mixed effects model. When the goal is to make predictions about the haplotype effects, this model choice will not influence the results.

#### 2.2.1. Prior Distributions

We assigned penalised complexity (PC) prior distributions to the variance parameters and the autocorrelation parameter. PC priors are proper prior distributions developed by Simpson et al. (2017) that penalise increased complexity as measured by deviation from a simpler base model to avoid over-fitting. For a random effect with a variance parameter the base model has variance of this random effect zero. For the autoregressive model of order one we have assumed a base model with ρ = 1. We could have assumed a base model with ρ = 0, but it is more likely that phylogenetically similar haplotypes have similar effects than completely independent effects. The PC prior can be specified through a parameter *u* and a probability α which satisfy Prob(*x* > *u*_*x*_) = α_*x*_ for the parameter *x*. We emphasise that the parameter *u* here is not an element of the allele sequence matrix **U** mentioned above.

Although the precision matrix of the haplotype effects is specified with the conditional variance in Equation (10), the prior is specified for the marginal variance since we often have a better intuition for the marginal variance than for the conditional variance. Specifically, we specify the prior for the marginal standard deviation σ_*h*_*m*__, and assume the conditions *u*_σ_*h*_*m*___ > 0 and 0 < α_σ_*h*_*m*___ < 1. For the autocorrelation parameter we use the PC prior developed for stationary autoregressive processes (Sørbye and Rue, 2017) with base model at ρ = 1, and parameters satisfying −1 < *u*_ρ_ < 1 and $\sqrt{\left(1-u_{\rho }\right)/2}<\alpha_{\rho }<1$. We highlight that the prior by Sørbye and Rue (2017) was developed for a stationary autoregressive process with different model assumptions than the models presented in this paper. Ideally, the prior for the autoregressive parameter would be tailored to the haplotype network model.

### 2.3. Inference and Evaluation

We describe the used method for statistical inference—the Integrated nested Laplace approximations (INLA)—and the methods used for evaluating model fit in the simulation study.

#### 2.3.1. Inference

All models in this study fit in the framework of hierarchical latent Gaussian models, which makes INLA (Rue et al., 2009) a suitable choice to perform inference as implemented in the R (R Core Team, 2018) package INLA (available at www.r-inla.org). We give a brief introduction to latent Gaussian models and how INLA is used to approximate the marginal posterior distributions in such models. For an in-depth description of INLA (see Rue et al., 2009, 2017; Blangiardo and Cameletti, 2015).

The class of latent Gaussian models includes several models, for example generalised linear (mixed) models, generalised additive (mixed) models, spline smoothing methods, and the models presented in this article. Latent Gaussian models are hierarchical models where observations ***y*** are assumed to be conditionally independent given a latent Gaussian random field ***x*** and hyper-parameters ***θ***_1_, meaning $p\left(y|x,\theta_{1}\right)\sim \Pi_{i\in I}p\left(y_{i}|x_{i},\theta_{1}\right)$. The latent field ***x*** includes both fixed and random effects and is assumed to be Gaussian distributed given hyper-parameters ***θ***_2_, that is $p\left(x|\theta_{2}\right)\sim N\left(\mu \left(\theta_{2}\right),\Sigma \left(\theta_{2}\right)\right)$. The parameters ***θ*** = (***θ***_1_, ***θ***_2_) are known as hyper-parameters and control the Gaussian field and the likelihood for the data. These are usually variance parameters for simple models, but can also include other parameters, for example the ρ parameter in the autoregressive model. We must also assign prior distributions to the hyper-parameters to completely specify the model.

The main aim of Bayesian inference is to estimate the marginal posterior distribution of the variables of interest, that is, *p*(θ_*j*_|***y***) for hyper-parameters and *p*(*x*_*i*_|***y***) for the latent field. INLA computes fast approximations to these densities with high accuracy. The INLA methodology is based on numerical integration and utilising Markov properties. Hence, for the computations to be both fast and accurate, the latent Gaussian models have to satisfy some assumptions. The number of non-Gaussian hyper-parameters ***θ*** should be low, typically less than 10, and not exceeding 20. Further, the latent field should not only be Gaussian, it must be a Gaussian Markov random field. The conditional independence property of a Gaussian Markov random field yields sparse precision matrices which makes computations in INLA fast due to the use of efficient algorithms for sparse matrices. Lastly, each observation *y*_*i*_ should depend on the latent Gaussian field only through one component *x*_*i*_.

The R package INLA is run using the inla() function with three mandatory arguments: a data frame or stack object containing the data, a formula much like the formula for the standard lm() function in R, and a string indicating the likelihood family. Prior distributions for the hyper-parameters are specified through additional arguments. Several tools to manipulate models and likelihoods exist as described in tutorials at www.r-inla.org and the books by Blangiardo and Cameletti (2015), and Krainski et al. (2018). In the Supplementary Material (Supplemental 1), we have included a script showing how we simulated the data from the haplotype network model and how we fitted the model to the data.

#### 2.3.2. Evaluation of Model Performance

We evaluated the model fit with the continuous rank probability score (CRPS) (Gneiting and Raftery, 2007). The CRPS is a proper score which takes into account the whole posterior distribution. It is negatively oriented, so the smaller the CRPS the closer the posterior distribution is to the true value. The full Bayesian posterior output from inla() for these models are mixtures of Gaussians, for which there is no closed form expression for CRPS. The mixtures here are similar to plain Gaussians, so we approximate the exact CRPS with the Gaussian CRPS using only the posterior mean and variances provided in the results.

We calculated the CRPS for estimated haplotype effects with the IH, HN and mutation models. To ease the comparison we have then calculated a relative CRPS (RCRPS) score as the log of the ratio between the averages of the CRPS from the HN model and IH model, and correspondingly for the mutation model relative to the IH model. The score is computed as:

$$\begin{array}{l} \log\left(\frac{\sum_{i=1}^{n}\text{CRPS}{\left({\hat{h}}_{i}\right)}_{HN}}{\sum_{i=1}^{n}\text{CRPS}{\left({\hat{h}}_{i}\right)}_{IH}}\right), \end{array}$$

where CRPS($\hat{h}$_*i*_)_*HN*_ is the CRPS of the posterior distribution for haplotype effect *h*_*i*_ with the HN model. We will refer to this score as the RCRPS.

We also calculated the root mean square error (RMSE) between the mean posterior haplotype effect and true haplotype effects, but the results for the relative RMSE and RCRPS were qualitatively the same. We therefore only present the RCRPS results.

In addition to comparing the haplotype estimates, we compared the estimated mutation effects from the HN model and the mutation model, using the RCRPS (HN model vs. mutation model). Although the HN model estimates the haplotype effects ***h***, we can obtain mutation effects via ***v*** = (**U**^*T*^**U**)^−1^**U**^*T*^***h***. We could also obtain mutation effects through linear combinations of haplotype effects.

### 2.4. Simulation Study

To test the proposed HN model, we first used simulated data. Here, we present data simulated from two different models—the HN model with varying degree of autocorrelation, and a more realistic mutation model where only some mutations have causal effect. We also present the models that were fitted to the simulated data, and how the model fit was evaluated. In the Supplementary Material (Supplemental 1), we provide an R script and the data file to simulate from and fit the haplotype network model.

#### 2.4.1. Simulation From the Haplotype Network Model

**Figure 2:**
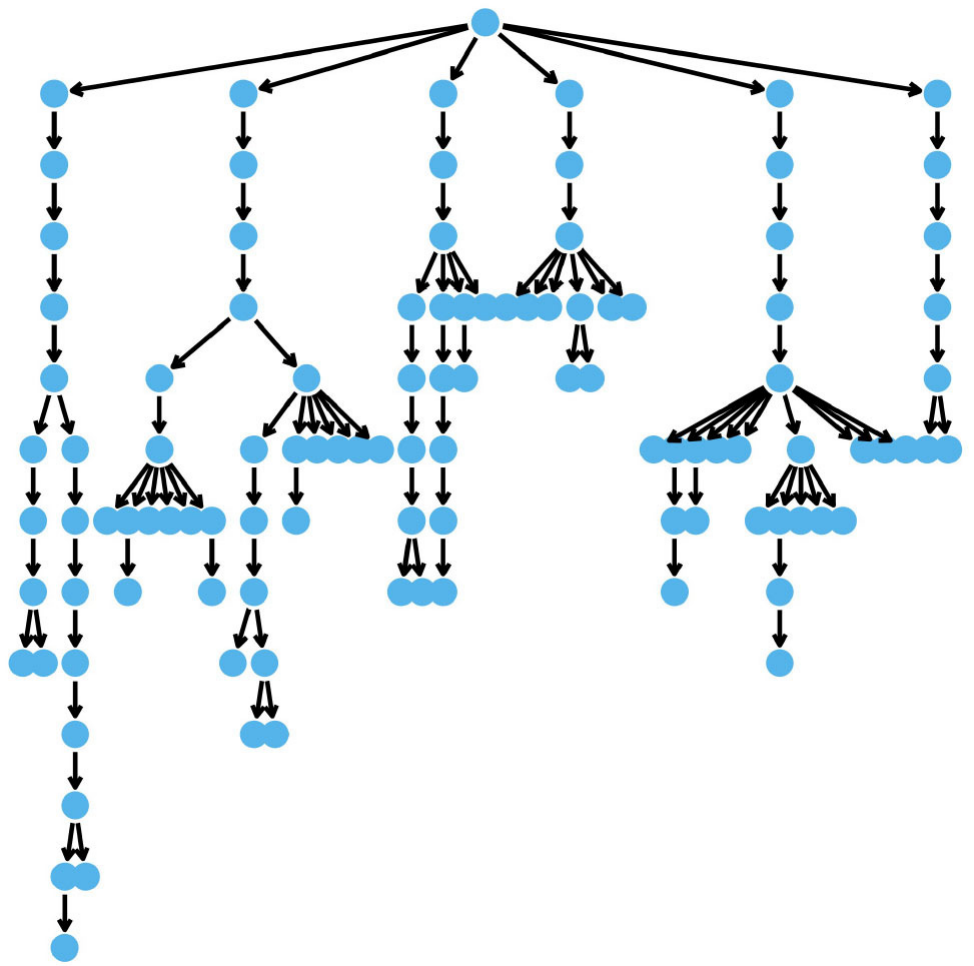
The DAG describing the phylogeny of simulated haplotypes.

We used the coalescent simulator msprime (Kelleher et al., 2016) to simulate the phylogeny shown in Figure 2 with *n* = 107 unique haplotypes. A script showing how this was performed is provided in the Supplementary Material (Supplemental 1) We then simulated phenotypes ***y*** for *p* = 400 individuals from the model:

(13) $$\begin{array}{l} y_{p\times 1}=Z_{p\times n}h_{n\times 1}+e_{p\times 1}, \end{array}$$

where $h\sim N\left(0,{\text{V}}_{h}\left(\rho ,\sigma_{h_{c}}^{2}\right)\right)$ with ${\text{V}}_{h}\left(\rho ,\sigma_{h_{c}}^{2}\right)$ built from the DAG describing the phylogeny (Figure 2 Equation 5), and $e\sim N\left(0,\text{I}\sigma_{e}^{2}\right)$.

We tested 15 parameter sets, from weak to strong haplotype effect dependency, and from low to high residual variance relative to the conditional haplotype variance:

$$\begin{array}{l} \rho =\left\{0.1,0.3,0.5,0.7,0.9\right\},\\ \sigma_{e}^{2}/\sigma_{h_{c}}^{2}=\left\{0.5,1,2\right\}. \end{array}$$

We simulated a haploid system for simplicity, so the incidence matrix **Z** was a zero matrix with a single 1 on each row indicating which individuals had which haplotype. We were particularly interested in estimating the haplotype effect with few or no direct phenotype observations. This is the extreme scenario where the haplotype network model could be beneficial. To achieve this, we designed the incidence matrix to create two different scenarios. In the first scenario, all haplotypes had associated phenotype observation, but some haplotypes only had one observation. We assigned a random sample of 15% of the haplotypes only to one individual each and the rest of the haplotypes randomly to the remaining individuals. In the second scenario, some haplotypes did not have phenotype observations. We selected a random sample of 15% of the haplotypes that did not have phenotype observations and assigned phenotype observations to the rest of the haplotypes. The values of the simulated observations ranged between −7.2 and 7.3.

#### 2.4.2. Simulation From the Mutation Model

We also simulated haplotype effects from a mutation model using the same phylogeny as in the previous section, shown in Figure 2, and using *p* = 400 individuals. For the 107 unique haplotypes we had 106 mutations in the haplotypes. We used the variants at these mutations to simulate haplotype effects and phenotypes according to the model:

(14) $$\begin{array}{l} y_{p\times 1}=Z_{p\times n}h_{n\times 1}+e_{p\times 1}, \end{array}$$

where ***h*** = **U**_*n*×106_***v***_106 ×1_, ***v*** was the mutation effect, **U** a matrix containing ancestral (reference) alleles coded as zero and alternative alleles coded as 1, and $e\sim N\left(0,\text{I}\sigma_{e}^{2}\right)$. We sampled the mutation effect *v* from:

$$\begin{array}{l} v=\{\begin{array}{c} N\left(0,\sigma_{v}^{2}\right),\text{with probability}\lambda \\ 0,\text{with probability}\left(1-\lambda \right) \end{array} \end{array}$$

where we chose $\sigma_{v}^{2}$ so that the empirical variance of ***h***, Var(***h***), was 1.

Again, we tested 15 parameter sets, from few to many causal variants, and from low to high residual variance relative to empirical haplotype variance:

$$\begin{array}{l} \lambda =\left\{0.1,0.3,0.5,0.7,0.9\right\},\\ \sigma_{e}^{2}/\text{Var}\left(\text{h}\right)=\left\{0.5,1,2\right\}. \end{array}$$

We again simulated haploid individuals, so the incidence matrix **Z** was a zero matrix with a single 1 on each row indicating which individuals had which haplotype. The incidence matrix was designed to create the same scenarios as for the data simulated from the HN model in section 2.4.1. The values of the simulated observations ranged between −8.4 and 8.9.

#### 2.4.3. Models Fitted to the Simulated Data

We fitted the HN model, IH model and the mutation model to the simulated data:

(15) $$\begin{array}{l} y_{p\times 1}=Z_{p\times n}h_{n\times 1}+e_{p\times 1}, \end{array}$$

where ***h*** was assumed to be distributed according to:

$h\sim N\left(0,{\text{V}}_{h}\left(\rho ,\sigma_{h_{c}}^{2}\right)\right)$ for the HN model,
$h\sim N\left(0,\text{I}\sigma_{I}^{2}\right)$ for the IH model and
***h*** = **U***v*, $\nu \sim N\left(0,\text{I}\sigma_{v}^{2}\right)$ for the mutation model.

The residual effect was $e\sim N\left(0,\text{I}\sigma_{e}^{2}\right)$. We used PC priors for the ρ parameters with *u*_ρ_ = 0.7 and α_ρ_ = 0.8, and for all variance parameters with *u* = 0.1 and α = 0.8.

#### 2.4.4. Evaluation

For each parameter set, we performed the same experiment 50 times. In 4% of the experiments when the data was simulated from the HN model, the optimisation method with the HN model did not converge. We report results only for cases where all models were successfully fitted. There was no trend for any parameter set causing the inference method to break down.

Since we created different scenarios for how phenotype observations were distributed among the haplotypes, we stratified the results for haplotype effects based on how many times a haplotype was phenotyped. For the first scenario, where some haplotypes were phenotyped either once or multiple times, we have computed the RCRPS for these two groups separately. For the second scenario, where some haplotypes were not phenotyped, we present the RCRPS only for haplotypes that were not phenotyped. In both cases, RCRPS less than zero indicates that the HN/mutation model was better than the IH model on average. We present the RCRPS for estimated mutation effects only for the mutation model simulation, because the true mutation effects were not generated when simulating from the haplotype network model.

### 2.5. Case Study: Mitochondrial Haplotypes in Cattle

We present a case study using the haplotype network model to estimate the effect of mitochondrial haplotypes on milk yield in cattle. We first briefly describe the data and then the fitted model.

#### 2.5.1. Data

We demonstrate the use of the haplotype network model with a case study estimating the effect of mitochondrial haplotypes on milk yield in cattle from Brajković (2019). We chose this case study because mitochondrial haplotypes are passed between generations without recombination and are as such a good case for the haplotype network model. The phenotyped data comprised of information about the first lactation milk yield, age at calving, county, herd-year-season of calving for 381 cows. Additionally, the data comprised of pedigree information with 6,336 individuals (including the 381 cows) and information about mitochondrial haplotypes (whole mitogenome with 16,345 bp) variation between maternal lines in the pedigree. We inferred the mitochondrial haplotypes by first sequencing mitogenome, aligning it to the reference sequence and calling 363 single-nucleotide mutations as described in detail in Brajković (2019). We used PopART (Leigh and Bryant, 2015) to build a phylogentic network of mitochondrial haplotypes. For simplicity we used the median-joining method to show that the haplotype network model can be fit to the output of a standard phylogentic method. In this process we assumed that the ancestral alleles were the most frequent alleles. The phylogeny contained 63 unique mitochondrial haplotypes each separated by one mutation. Of the 63 haplotypes only 16 haplotypes were observed in the 381 phenotyped cows. There were five haplotypes that did not have a parent haplotype, meaning we treated them as a “starting” haplotype in the haplotype network model.

#### 2.5.2. Model

Let ***h***_*n*×1_ be the effect of the *n* = 63 mitochondrial haplotypes, and let ***y***_*p*×1_ be the phenotypes of the *p* = 381 cows. We fitted the following model to centred and scaled phenotypes:

$$\begin{array}{l} y_{p\times 1}={\text{X}}_{p\times r}\beta_{r\times 1}+c_{p\times 1}+a_{p\times 1}+Z_{p\times n}h_{n\times 1}+e_{p\times 1} \end{array}$$

where β~N(0,I1000) contained effects of age at calving as a continuous covariate effect and county as a categorical covariate effect with corresponding design matrix **X**, $c\sim N\left(0,\text{I}\sigma_{c}^{2}\right)$ was the random effect of herd-year-season of calving (contemporary group), $a\sim N\left(0,A\sigma_{a}^{2}\right)$ was additive genetic effect for the whole nuclear genome with the covariance coefficient matrix *A* derived from the pedigree (Henderson, 1976; Quaas, 1988), and lastly the mitochondrial haplotype effects were fitted with the haplotype network model $h\sim N\left(0,{\text{V}}_{h}\left(\rho ,\sigma_{h_{c}}^{2}\right)\right)$ with the covariance matrix ${\text{V}}_{h}\left(\rho ,\sigma_{h_{c}}^{2}\right)$ derived from the phylogeny and using the expanded model that accommodates multiple parental haplotypes from section 2.1.3. We assumed that residuals were distributed as $e\sim N\left(0,\text{I}\sigma_{e}^{2}\right)$. We assigned PC priors to the ρ parameter with *u*_ρ_ = 0.7 and α_ρ_ = 0.8 and to the $\sigma_{h_{m}}^{2}$ parameter with *u*_σ_*h*_*m*___ = 0.1 and α_σ_*h*_*m*___ = 0.3, and to all remaining variance parameters with *u*_σ_*__ = 0.1 and α_σ_*__ = 0.8.

## 3. Results

We present results from the simulation study testing the behavior of the haplotype network model and the case study estimating the effect of mitochondrial haplotypes on milk yield in cattle. In the results from the simulation study, we present the RCRPS between the haploptype network (HN) model and the independent haplotype (IH) model, and between the mutation model and the IH model for the different parameter sets. In the results from the case study, we present the mean and standard deviation of the posterior mitochondrial haplotype effects mapped onto the phylogenetic network, and posterior estimates for the hyper-parameters.

### 3.1. Simulation Study

#### 3.1.1. Simulation From the Haplotype Network Model

We start by considering the results with the data simulated from the HN model from section 2.4.1 that were fitted with the models from section 2.4.3.

**Figure 3:**
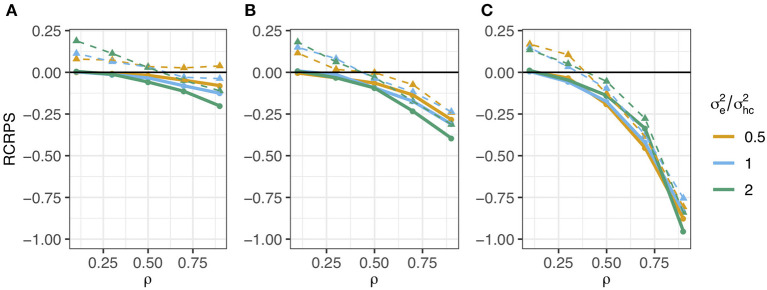
RCRPS (smaller values indicate that the HN or mutation models, respectively, are better than the reference IH model) between the HN model and the IH model (solid line) and between the mutation model and the IH model (dashed line) for data simulated from the HN model with varying ρ parameter and ratio between the residual $\sigma_{e}^{2}$ and conditional haplotype variance $\sigma_{h_{c}}^{2}$. The three panels show RCRPS for the haplotypes that were observed in **(A)** several phenotyped individuals, **(B)** only one phenotyped individual, and **(C)** were not observed in a phenotyped individual.

The RCRPS (smaller values indicate that the HN or mutation models, respectively, are better than the reference IH model) is presented in Figure 3. This figure has three panels denoting haplotypes that were observed in (Figure 3A) several phenotyped individuals, (Figure 3B) only one phenotyped individual and (Figure 3C) were not observed in a phenotyped individual. The full lines show the RCRPS between the HN model and the IH model, while the dashed lines show the RCRPS between the mutation model and the IH model. Along the *x*-axis the autocorrelation parameter ρ for the simulated haplotype effects increases from weak to strong phylogenetic dependency, and the three colored lines indicate the amount of phenotypic variation due to residual relative to the variation from haplotype effects.

Figure 3 shows that (1) the HN model outperforms the IH model across a range of parameter values, (2) the HN model is more important for haplotypes with fewer phenotypic observations, (3) the HN model is more important for noisy phenotypic data, and (4) when haplotypes are more phylogenetically dependent, the HN model and the mutation model have similar performance. We go through each of these findings in detail.

The HN model outperforms the IH model for almost all 15 parameter sets. In all panels of Figure 3 almost all points with the full line are below zero, meaning that the HN model gave better estimates of haplotype effects than the IH model. When the haplotype dependency due to phylogeny was low, the RCRPS was around zero, meaning that the two models performed similarly, which was expected. As the phylogenetic dependency became stronger, the HN model improved relative to the IH model, as seen from the decreasing RCRPS as ρ approaches 0.9.

The improvement in CRPS with the HN model relative to the IH model increased when haplotypes were observed in a smaller number of phenotyped individuals. This is indicated by the decreasing RCRPS when we compare panels (A), (B), and (C) in Figure 3. The decrease in RCRPS was the largest in Figure 3C followed by Figure 3B and Figure 3A. This means that modelling phylogenetic dependency between haplotypes is most useful when there are some haplotypes with few phenotypic observations, or if we want to predict the effect of new haplotypes. Especially for haplotypes that do not have a direct link to observed phenotypes, the IH model is not useful, because it assigns the average effect of haplotypes with direct link to observed phenotypes to haplotypes without such links, whereas the HN model can assign the haplotype effect based on a phylogenetic network. When the haplotype effects had low phylogenetic dependency (ρ is low), there was not much difference in RCRPS between the three panels.

The improvement with the HN model relative to the IH model increased when the phenotypic data was noisier. In Figures 3A,B, the RCRPS was lower with larger residual variance. This indicates that the HN model does a better separation of the environmental and genetic sources of variation than the IH model. We did not observe the same in Figure 3C, because the IH model performed equally poorly in predicting new haplotypes regardless of the amount of residual variance. The HN model on the other hand, performed slightly better as there was less variation due to residual effects for some values of ρ and similar for other values of ρ compared to the IH model.

As haplotypes became phylogentically more dependent with the increasing ρ, the HN model and the mutation model performed similarly. In all panels the dashed lines indicate a worse fit for the mutation model than for the IH model and HN model when ρ was low. When ρ increased, the mutation model improved relative to the IH model, but not better than the HN model.

#### 3.1.2. Simulated Data From the Mutation Model

Now, we consider the results with the haplotype effects simulated from a more realistic mutation model in section 2.4.2, and fitted with the models from section 2.4.3. Here we varied the probability of mutations having a causal effect λ and we present results using only λ = 0.1, since the results were qualitatively similar for all tested λ values.

**Figure 4:**
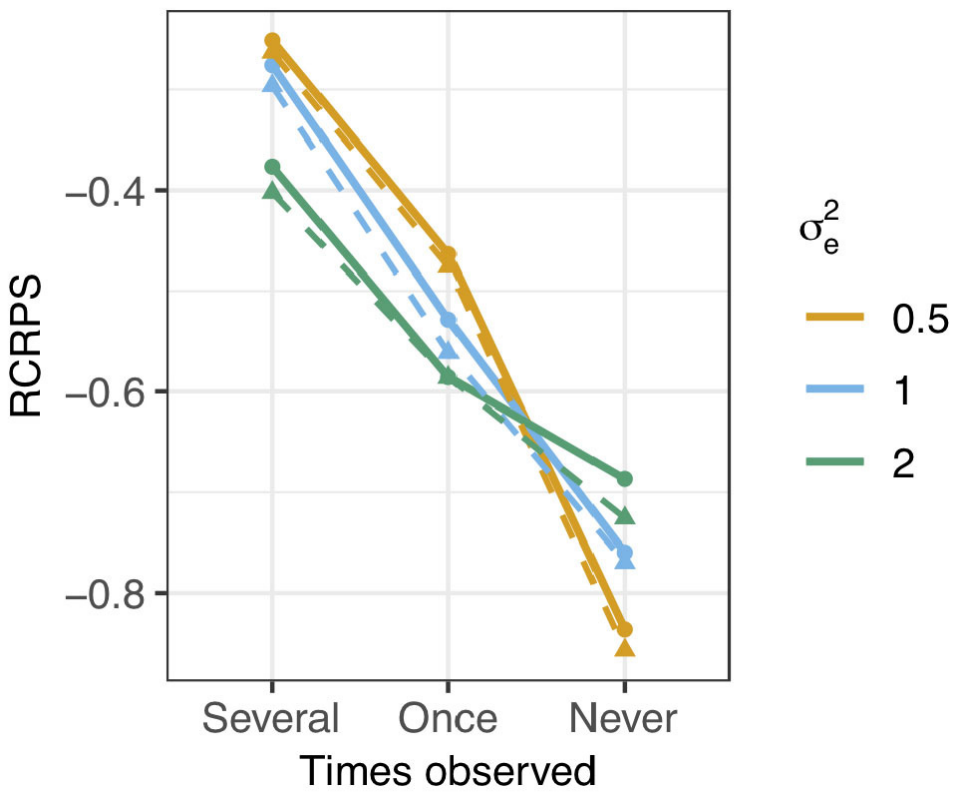
RCRPS (smaller values indicate that the HN or mutation models, respectively, are better than the reference IH model) between the HN model and the IH model (solid line) and between the mutation model and the IH model (dashed line) for data simulated from the mutation model with varying residual variance $\sigma_{e}^{2}$ and empirical haplotype variance 1 [Var(***h***) = 1]. The three scenarios show RCRPS for the haplotypes that were observed in (Several) several phenotyped individuals, (Once) only one phenotyped individual, and (Never) were not observed in a phenotyped individual.

The RCRPS is presented in Figure 4 for the three different levels of phenotype observations per haplotype and three different values of residual variance relative to the empirical haplotype variance which was always 1. The full lines show the RCRPS between the HN model and the IH model, while the dashed lines show the RCRPS between the mutation model and the IH model.

In general, the results align with the results from the previous section except for the mutation model; (1) the HN model outperforms the IH model, (2) the HN model is more important for haplotypes with few phenotypic observations, (3) the HN model is more important for noisy phenotypic data and (4) the mutation model was marginally better than the HN model in estimating haplotype effects. We go through each of the findings in detail.

The HN model outperformed the IH model for all tested parameter sets. In Figure 4, all RCRPS values, are well below zero. For haplotypes observed in several or one phenotyped individual, the RCRPS was lower than what was seen in Figures 3A,B. For haplotypes with no direct links to phenotype observations, the RCRPS was not improving as much as seen in Figure 3C.

The improvement with the HN model relative to the IH model increased with fewer phenotype observations per haplotype. The RCRPS in Figure 4 is lowest for haplotypes with no direct links to phenotype observations, second lowest for haplotypes with one direct link to a phenotype observation, and highest for haplotypes that were observed in several phenotyped individuals.

The improvement with the HN model relative to the IH model increased with increasing residual variation. In Figure 4, the RCRPS for haplotypes observed in several or one phenotyped individual decreases with increasing residual variance. This was again not the case for haplotypes with no direct links to phenotype observations. As mentioned in the previous section, the IH model is predicting new haplotypes equally poorly irrespective of the residual variance. The HN model on the other hand, improves the prediction of new haplotypes when the phenotypic data is less noisy.

The mutation model was marginally better than the HN model in estimating haplotype effects. The dashed lines in Figure 4 indicate the RCRPS between the mutation model and the IH model, and the full lines indicate the RCRPS between the HN model and the IH model. The dashed lines and full lines follow each other closely, and the dashed lines are slightly lower than the full lines, indicating that the mutation model was slightly better than the HN model, although not by much.

In Table 2, we present the average RCRPS between the HN model and the mutation model for the estimated mutation effects. This table has the RCRPS for the two scenarios where either all haplotypes had associated phenotype observation, or most haplotypes had associated phenotype observation and the rest did not, with different proportions of mutations with causal effect and for different residual variance. RCRPS above zero indicate that the mutation model had better CRPS, and averages below zero indicate that the HN model had better CRPS. Overall the difference between the two models is small. The mutation model had the best performance when there were few causal mutations, and the HN model had the best performance when there were many causal mutations.

**Table 2:** RCRPS between the HN model and the mutation model for mutation effects by different values of residual variance $\sigma_{e}^{2}$, proportion of causal mutations and for the two scenarios where either all or most haplotypes have direct links to observed phenotypes.

**Prop. of causal mut.**	**All observed**	**Most observed**
$\sigma_{e}^{2}=0.5$		
0.1	0.060	0.071
0.3	0.019	0.025
0.5	−0.002	−0.004
0.7	−0.019	−0.021
0.9	−0.027	−0.029
$\sigma_{e}^{2}=1$		
0.1	0.123	0.111
0.3	0.043	0.037
0.5	0.004	0.000
0.7	−0.024	−0.022
0.9	−0.041	−0.034
$\sigma_{e}^{2}=2$		
0.1	0.168	0.214
0.3	0.067	0.101
0.5	0.006	0.018
0.7	−0.025	−0.026
0.9	−0.042	−0.048

### 3.2. Case Study: Mitochondrial Haplotypes in Cattle

We present results for the case study of estimating the effect of mitochondrial haplotypes on milk yield in cattle presented in section 2.5. We present the posterior mean and standard deviation for the effect of mitochondrial haplotypes mapped onto the phylogeny, the posterior distribution for the autocorrelation parameter ρ, and the mean and 95% confidence interval of the posterior variances in the model.

In summary, the results show (1) that there was sharing of information between the mitochondrial haplotypes, (2) that haplotypes without a direct link to observed phenotyopes were estimated with larger uncertainty, (3) indications of strong phylogenetic dependency between the haplotypes, and (4) a significant proportion of the total phenotypic variation explained by mitochondrial haplotypes.

**Figure 5:**
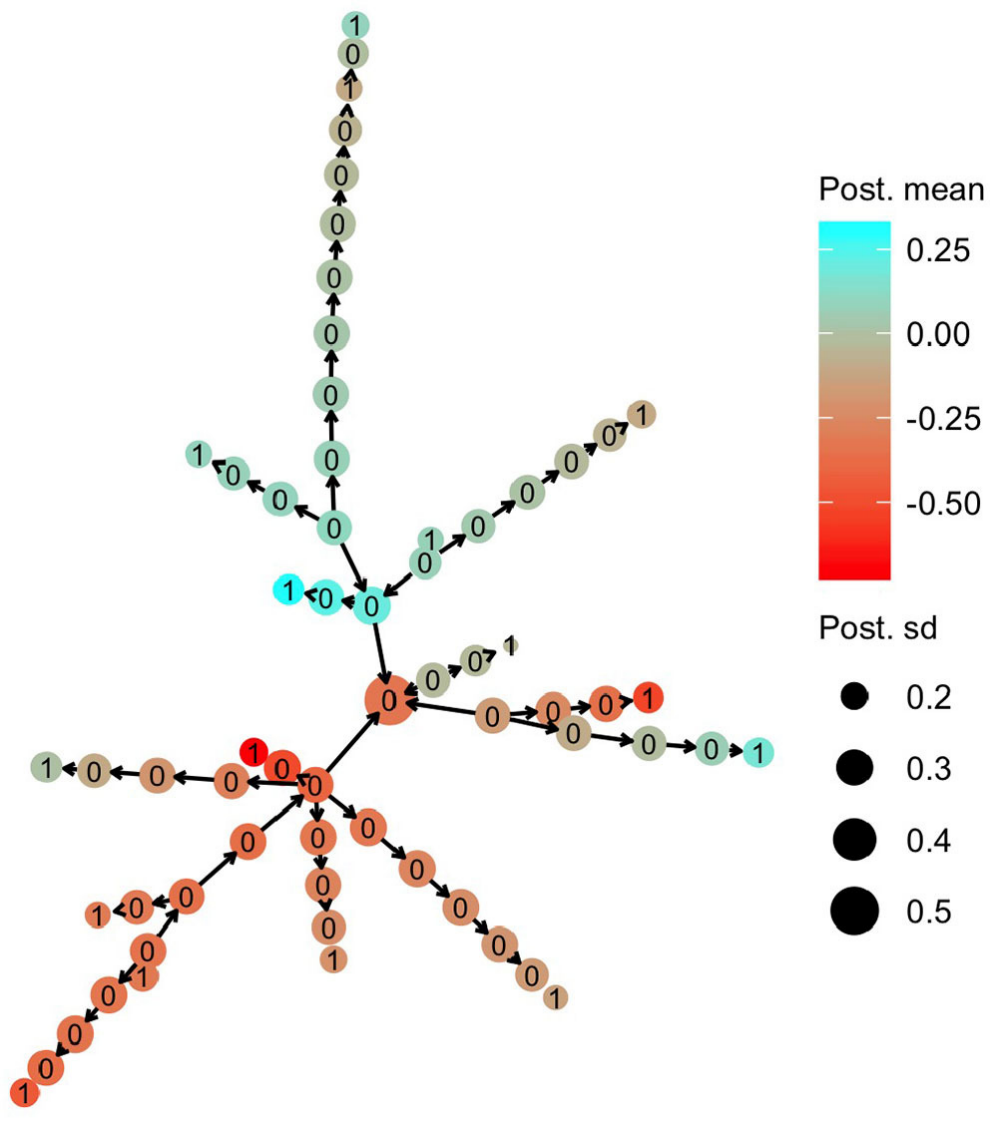
Posterior mean and standard deviation for mitochondrial haplotype effects on milk yield in cattle. Posterior means are denoted with node color, while posterior deviations are denoted by the node size. The numbers on each haplotype node indicate if the haplotype had a direct link to the observed phenotype (1) or not (0).

The HN model enabled sharing of information from the haplotypes that had a direct link with observed phenotypes to the other haplotypes. In Figure 5, we present the posterior mean for the effect of mitochondrial haplotypes with node color. Haplotype effect estimates are similar for phylogenetically similar haplotypes, meaning that there was sharing of information between the haplotypes, even though haplotypes that had direct links with phenotype observations (nodes labelled with 1) were separated from each other with a substantial number of mutations.

Haplotypes without direct links to observed phenotypes were estimated with larger uncertainty. In Figure 5, we present the posterior standard deviation for the effect of mitochondrial haplotypes with node size. Haplotypes with direct links to observed phenotypes (nodes labelled with 1) have smaller posterior standard deviation than the other haplotypes (nodes labelled with 0). The posterior standard deviation decreased slightly as the haplotypes without direct links were closer (in number of mutations) to the haplotypes with direct links, which was expected.

**Figure 6:**
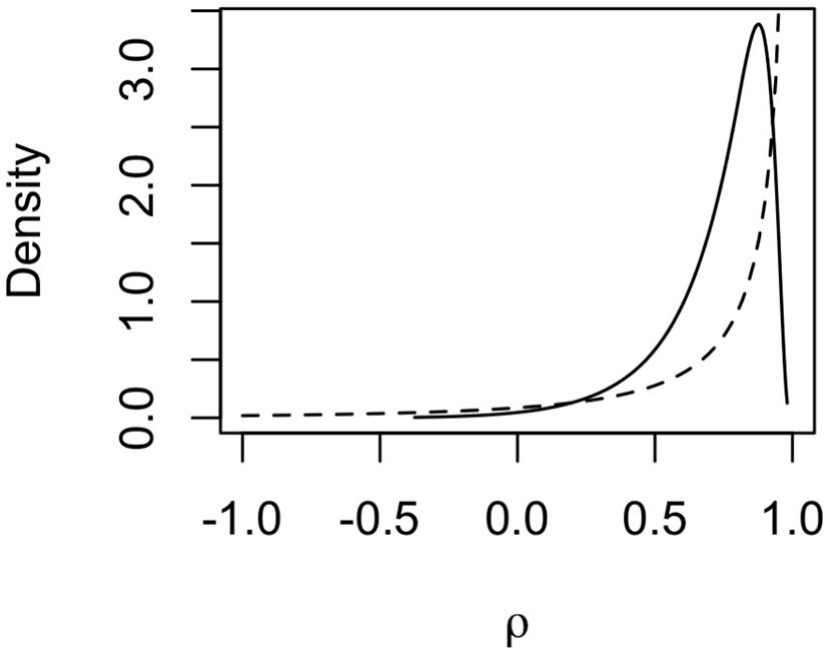
Prior (dashed line) and posterior (solid line) distribution for the autocorrelation parameter ρ for mitochondrial haplotype effects on milk yield in cattle.

The posterior distribution for the autoregression parameter ρ indicated strong dependency between haplotype effects. The posterior distribution (full line) of ρ is shown in Figure 6 together with the prior distribution (dashed line). The mode of the distribution lies around 0.85, and the mean lies around 0.73, indicating that neighboring haplotypes had similar effects, which is related to the sharing of information between haplotypes seen in Figure 5. We also note that the posterior distribution shifted to slightly lower values of ρ than the prior distribution. This means that the data contained information that the model could learn from.

A significant amount of the total phenotypic variation was explained by the mitochondrial haplotypes. In Table 3, we present the posterior mean and 95% confidence interval of each variance component in the model, and how much of the total variation in the model ($\sigma_{c}^{2}+\sigma_{a}^{2}+\sigma_{h_{m}}^{2}+\sigma_{e}^{2}$) was explained by each variance component. The posterior distribution of the conditional haplotype variance was obtained by computing $\sigma_{h_{c}}^{2}=\sigma_{h_{m}}^{2}\left(1-\rho^{2}\right)$, using 10,000 samples from the posterior distributions of the marginal haplotype variance and the autocorrelation parameter. We see that the marginal haplotype variance $\sigma_{h_{m}}^{2}$ and conditional haplotype variance $\sigma_{h_{c}}^{2}$ is smaller compared to the additive genetic variance $\sigma_{a}^{2}$, and the residual variance $\sigma_{e}^{2}$. This was expected as the mitogenome (~ 1 × 16*Kbp*) is much smaller than the nuclear genome (~ 2 × 3*Gbp*). In the light of this difference we can say that mitochondrial haplotypes captured a significant amount of phenotypic variation. The variance for the random effect of herd-year-season of calving $\sigma_{c}^{2}$ was also smaller compared to $\sigma_{a}^{2}$ and $\sigma_{e}^{2}$.

**Table 3:** Posterior mean, 95% confidence interval (CI) for variance parameters, and the proportion of variation explained by each variance component for the case study estimating mitochondrial haplotype effects on milk yield in cattle.

**Variance parameter**	**Mean**	**95% CI**	**Prop. of variance explained**
$\sigma_{c}^{2}$	0.035	(0.005, 0.090)	0.047
$\sigma_{a}^{2}$	0.329	(0.194, 0.533)	0.444
$\sigma_{h_{m}}^{2}$	0.113	(0.033, 0.264)	0.152
$\sigma_{h_{c}}^{2}$	0.048	(0.007, 0.154)	0.065
$\sigma_{e}^{2}$	0.265	(0.171, 0.416)	0.357

*$\sigma_{c}^{2}$, variance of contemporary group effects; $\sigma_{a}^{2}$, variance of nuclear-genome additive effects; $\sigma_{h_{m}}^{2}$, marginal variance of mitogenome haplotype effects; $\sigma_{h_{m}}^{2}$, conditional variance of mitogenome haplotype effects; $\sigma_{e}^{2}$, variance of residuals*.

It should be noted that this is a small data set with few haplotypes with direct links to observed phenotypes, which means that the posterior standard deviations for haplotype effects were relatively large. This also causes posterior estimates to be strongly influenced by the prior distributions, especially the posterior for ρ which we can see in Figure 6. However, we still chose to assign an informative prior to ρ, since it is expected that most mutations have no causal effect and that phylogenetically similar haplotypes have similar effects.

### 3.3. Computation Time

The models were run on a computation server with Linux operating system, 24 cores (4x6 core 2.66 GHz Intel Xeon X7542) and 256 GB memory, fitting up to seven models in parallel. The R version used to produce the results was 3.6.0, and the INLA package version was 18.07.12. INLA was allowed to use as many threads as were available.

In the simulation study, the average computation time was 359.3 s with the HN model, 3.4 s with the IH model, and 4.4 s with the mutation model when the data were simulated from the haplotype network model. When the data were simulated from the mutation model, the average computation time was 310.4 s for HN model, 3.2 s for the IH model and 1.4 s for the mutation model. For the case study with mitochondrial haplotypes, the computation time with the HN model was 119 s.

## 4. Discussion

The objective of this paper was to propose a hierarchical model that leverages haplotype phylogeny to improve the estimation of haplotype effects. We have presented the haplotype network model, evaluated it using simulated data from two different generative models, and applied it in a case study of estimating the effect of mitochondrial haplotypes on milk yield in cattle. We highlight three points for discussion in relation to the proposed haplotype network model: (1) the importance of the haplotype network model, (2) future development and possible extensions and (3) limitations.

### 4.1. The Importance of the Haplotype Network Model

We see three important advantages of the haplotype network model; the ability to share information between related haplotypes, computational advantages when modelling a single region of a genome, and the potential to capture background specific mutation effects.

The haplotype network model utilises phylogenetic relationships between haplotypes and with this improves estimation of their effects. From the simulation study, we saw the importance of this information sharing when there is limited information per haplotype. In the haplotype network model the autocorrelation parameter ρ and the conditional variance parameter $\sigma_{h_{c}}^{2}$ reflect the covariance between effects of phylogenetically similar haplotypes. As the autocorrelation approaches 1, haplotype effects become more dependent. Further, if the conditional variance is small the large dependency and small deviations lead to similar effects for phylogenetically similar haplotypes, suggesting that mutations separating the haplotypes have very small or no effect compared to other shared mutations between haplotypes. If on the other hand conditional variance is large, the large dependency and large deviations lead to haplotype effects that change rapidly along the phylogeny, suggesting that mutations separating the haplotypes have large effects. On the other hand, if the autocorrelation parameter approaches 0, the covariance between effects of phylogenetically similar haplotypes is decreasing, suggesting that haplotypes should be modelled independently.

The three extreme scenarios of hyper-parameter values could denote three real cases. The first case with high autocorrelation and small conditional variance could reflect a situation where the whole haplotype sequence would be used to build a phylogeny and since most mutations do not have a causal effect, but some do, it is expected that similar haplotypes will have similar effects with small differences between the haplotypes. The second case with high autocorrelation and large conditional variance could reflect the situation when the number of causal mutations would be high compared to all mutations (because only such mutations are analysed) and therefore change of effects along the phylogeny would be larger. The third scenario with no autocorrelation could reflect the situation where phylogeny does not correlate with phenotype change.

As mentioned in the introduction, modelling phenotypic variation as a function of haplotype variation has extensive literature (Templeton et al., 1987; Balding, 2006; Thompson, 2013; Morris and Cardon, 2019). The prime motivation for this work is the recent growth in the generation of large scale genomic data sets and methods to build phylogenies (Kelleher et al., 2019). We aimed to develop a general model that could exploit phylogenetic relationships between haplotypes in a computationally efficient way. The computational benefits come from the sparse precision matrix ${\text{V}}_{h}^{-1}$, which is due to the conditional independence structure encoded in the DAG of a network of haplotypes (Rue and Held, 2005). The computational benefits are not critical when the number of haplotypes is small. In that case the matrix **V**_*h*_ is small and easy to invert, though for the autoregressive model we would have to invert it many times during the estimation procedure due to dependency on the autocorrelation parameter. However, it is better to avoid inversions if possible because it can lead to numerical errors and loss of precision (e.g., Misztal, 2016).

While the haplotype network model is different to the pedigree mixed model (Henderson, 1976; Quaas, 1988) (where we model the inheritance of whole genomes in a pedigree without (fully) observing the genomes) or the phylogenetic mixed model (Lynch, 1991; Pagel, 1999; Housworth et al., 2004; Hadfield and Nakagawa, 2010) (where we model the inheritance of whole genomes in a phylogeny without (fully) observing the genomes), the principles of conditional dependence between genetic effects and the resulting sparsity are the same (Rue and Held, 2005). The key difference of the haplotype network model is that it estimates the effect of observed haplotype sequences as compared to unobserved or partially observed inheritance of whole genomes in a pedigree or phylogeny. To improve the estimation of the haplotype effects we take into account the phylogenetic relationships. A similar model has also been used in spatial disease mapping (Datta et al., 2019), showing potential of this kind of model in several applications.

While the use of phylogenetic relationships might seem redundant if we know (most of) the haplotype sequence, the simulations showed that it improves estimation in most cases, even marginally compared to the mutation model where we directly model mutation effects. The haplotype network model can be seen as a hybrid between the mutation model (that models variation between the columns of a haplotype matrix) and the independent haplotype model (that models variation between the rows of a haplotype matrix). This hybrid view might improve genome-wide association studies (see reviews by Gibson, 2018; Simons et al., 2018; Morris and Cardon, 2019; Uricchio, 2019).

The haplotype network model has the potential to capture background specific mutation effects, which are effects observed when the effect of a mutation depends on other mutations present in an individual (e.g., Chandler et al., 2017; Steyn et al., 2019; Wojcik et al., 2019). If there are background specific mutation effects the haplotype effect differences will capture this, while a mutation model only estimates an average effect of a mutation across multiple backgrounds (haplotypes). However, we must point that the haplotype network model captures only local effects, that are due to interactions between mutations present on a haplotype (e.g., Clark, 2004; Liu et al., 2019). We have not evaluated how well the model captures background specific mutation effects in this study, and more simulations to a range of data are needed to evaluate this aspect.

### 4.2. Future Development and Possible Extensions

There is a number of areas for future development with the haplotype network model. We are looking into four areas: making the model more flexible in the number of mutations separating phylogentically similar haplotypes, modelling haplotype differences in a continuous way utilising branch lengths, incorporating biological information and phylogenetic aspects of haplotype relationships.

We have developed the haplotype network model by assuming the differences between similar haplotypes is due to one mutation to simplify model definition. However, in the observed data there might not be haplotypes that are separated for just one mutation. We handle this situation by inserting phantom haplotypes, to ensure that we do not model haplotypes as more similar than they actually are. The order of mutations in such situations is uncertain and a model could be generalised to account for these larger number of mutations between haplotypes. However, the current “one-mutation” difference model setup has a useful property of inferring the value of unobserved haplotypes and the sparse model definition does not increase computational complexity of the model.

The haplotype network model could be generalised to utilise time calibrated distances between haplotypes rather than using the number of mutations. The Ornstein-Uhlenbeck (OU) process is the continuous-time analogue of the autoregressive process of order one used in this study, and plays a major role in the analysis of the evolution of phenotypic traits along phylogenies (Lande, 1976; Hansen and Martins, 1996; Martins and Hansen, 1997; Paradis, 2014). Relatedly, if the autocorrelation parameter of the autoregressive process of order one is set to 1 we get the non-stationary discrete random walk process, whose continuous-time analogue is the Brownian process that is the basic model of phylogenetic comparative analysis (Felsenstein, 1988; Huey et al., 2019). There is a scope to improve computational aspects for these continuous models too by employing recent developments from the statistical analysis of irregular time-series (Lindgren and Rue, 2008).

In the haploptype network model presented in this study, the same autocorrelation parameter has been assumed for all mutations. However, the autocorrelation parameter could be allowed to vary as Beaulieu et al. (2012) did in the context of adaptive evolution. For example, different autocorrelation parameters for different types of mutations could incorporate biological information, which could combine the quantitative analysis of mutation and haplotype effects with molecular genetic tools such as Variant Effect Predictor (McLaren et al., 2016).

We have assumed that the phylogenetic network is given and described with a DAG. There is a large body of literature on inferring phylogenies in the form of strict bifurcating trees, more general trees or networks and recent developments in genomics are rapidly advancing the field (e.g., Anisimova, 2012; Puigbò et al., 2013; Schliep et al., 2017; Uyeda et al., 2018). The haplotype network model can work both with phylogenetic bifurcating and multifurcating trees and phylogenetic networks. The only condition is that we describe the haplotype relationships with a DAG, an output provided by many tools (e.g., Leigh and Bryant, 2015; Suchard et al., 2018; Kelleher et al., 2019). We have generalised the model construction to allow for network structures. This generalisation enables the model to describe haplotype relationships without paying attention to the directionality as long as there are no directed loops in the graph. The proposed model does not depend on which allele is ancestral, major or minor, but we believe that the most logical is to work with ancestral alleles as the starting point.

It is beneficial to know the order of mutations, and therefore which haplotypes are parental to other haplotypes, because this leads to a tree structure and a sparse precision matrix structure in the model (Rue and Held, 2005). An example of non-optimal sparsity can be seen in our case study. In Figure 5, the “central” haplotype with the largest uncertainty is modelled as a progeny haplotype of four surrounding haplotypes, which means that there is a dense 5 × 5 block in the precision matrix ${\text{V}}_{h}^{-1}$. The block is dense because the “central” haplotype is modelled as a function of the other four “parental” haplotypes. If however the “central” haplotype was used as the parental haplotype the 5 × 5 block would be sparse since all other haplotypes would be conditionally independent given the “central/parental” haplotype. The same applies also for the other parts of the haplotype network in Figure 5.

The haploptype network model could also work with probabilistic networks where edges have associated uncertainty (weights). By encoding such a network with a DAG, the edge weights can be used in model construction—for example, in the same way uncertain parentage is handled in pedigree models (Henderson, 1976). An alternative would be to construct a model for each possible realisation of a network, run separate models and combine haplotype estimates in the spirit of Bayesian model averaging.

### 4.3. Limitations

The haplotype network model also has some limitations that merit further development. We highlight three areas: is the haplotype network model necessary given that we can model mutation effects, Gaussian assumption and causal mutations, and modelling recombining haplotypes.

For the haplotype network model to achieve its full potential, the data need to have a certain structure. We saw from fitting the haplotype network model to a real data set, that having few haplotypes with direct links to observed phenotypes and many haplotypes without, lead to large uncertainty in estimated haplotype effects. We also saw from fitting simulated data, that the mutation model was slightly better at estimating the mutation effects than the haplotype network model, when the data were simulated from a mutation model, but the magnitude of difference was minimal. In the future, different data structures should be tested to find optimal scenarios, in order for the haplotype network model to achieve its full potential.

The haplotype network model assumes that the haplotype effects follow a Gaussian distribution. If all, or very many, of the haplotypes have the same effect, the distribution may be quite different from Gaussian, which breaks the model assumptions and perhaps other models should be proposed. Blomberg et al. (2019) describe the underlying theory behind the common Gaussian processes, such as Brownian motion and Ornstein-Uhlenbeck process, and present general methods for deriving new stochastic models, including non-Gaussian models of quantitative trait macroevolution. See also (Landis et al., 2012; Schraiber and Landis, 2015; Duchen et al., 2017; Bastide et al., 2020).

Scaling the haplotype network model to multiple recombining haplotype regions is challenging for two reasons. First, while phasing methods have improved substantially in the last years (Marchini, 2019), determining a recombination breakpoint is challenging due to a limited resolution to resolve exact locus where recombination occurred (Johnsson et al., 2020). Second, the sparsity of the haplotype network model comes from the sparsity of the precision matrix ${\text{V}}_{h}^{-1}$. In the extension for recombining haplotypes the sparsity in the prior is maintained also for multiple consequitive haplotype regions along a chromosome as shown in Equation (11) in section 2.1.4. However, the design matrices that link phenotype observations with multiple haplotype regions create dense cross-products in the system of equations as we increase the number of regions and the sparsity advantage is lost. To this end we are exploring alternative ways of formulating the haplotype network model following data structures in Kelleher et al. (2019), with the aim to improve upon the existing haplotype based genomic modelling of whole genomes (e.g., Villumsen et al., 2009; Hickey et al., 2013).

## Data Availability Statement

The datasets analysed in this article are not publicly available. Requests to access the datasets should be directed to Vladimir Brajkovic, vbrajkovic@agr.hr.

## Author Contributions

MLS, FL, and GG conceived and derived the haplotype network model. MLS, IS, and GG designed the analysis, and evaluated the results. MLS simulated data and performed all analyses. VB and VC-C provided case study data. MLS wrote the manuscript. FL, IS, and GG commented on and edited the manuscript. All authors have read and approved the final manuscript.

## Conflict of Interest

The authors declare that the research was conducted in the absence of any commercial or financial relationships that could be construed as a potential conflict of interest.

## Supplementary Material

Supplemental 1 R script and data file to simulate the DAG describing the phylogeny of simulated haplotypes, to simulate from the haplotype network model, and to fit the haplotype network model to the data.

## References

[B1] Anisimova M. (2012). Evolutionary Genomics Statistical and Computational Methods. New York, NY: Springer 10.1007/978-1-61779-585-5

[B2] Balding D. J. (2006). A tutorial on statistical methods for population association studies. Nat. Rev. Genet. 7:781. 10.1038/nrg1916 16983374

[B3] Basseville M. Benveniste A. Chou K. C. Golden S. A. Nikoukhah R. Willsky A. S. (1992). Modeling and estimation of multiresolution stochastic processes. IEEE Trans. Inform. Theory 38, 766–784. 10.1109/18.119735

[B4] Bastide P. Ho L. S. T. Baele G. Lemey P. Suchard M. A. (2020). Efficient bayesian inference of general gaussian models on large phylogenetic trees. arXiv [Preprint] arXiv:2003.10336.

[B5] Beaulieu J. M. Jhwueng D.-C. Boettiger C. O'Meara B. C. (2012). Modeling stabilizing selection: expanding the Ornstein-Uhlenbeck model of adaptive evolution. Evol. Int. J. Organ. Evol. 66, 2369–2383. 10.1111/j.1558-5646.2012.01619.x 22834738

[B6] Begum R. (2019). A decade of genome medicine: toward precision medicine. Genome Med. 11. 10.1186/s13073-019-0624-z. [Epub ahead of print]. 30819206 PMC6394063

[B7] Blangiardo M. Cameletti M. (2015). Spatial and Spatio-Temporal Bayesian Models with R-INLA. Chichester: John Wiley and Sons 10.1002/9781118950203

[B8] Blomberg S. P. Rathnayake S. I. Moreau C. M. (2019). Beyond brownian motion and the Ornstein-Uhlenbeck process: stochastic diffusion models for the evolution of quantitative characters. Am. Natural. 195, 000–000. 10.1086/706339 32017624

[B9] Brajković V. (2019). Utjecaj mitogenoma na svojstva mliječnosti goveda (Eng: Impact of mitogenome on milk traits in cattle) (Ph.D. thesis). University of Zagreb, Faculty of Agriculture, Zagreb, Croatia.

[B10] Chandler C. H. Chari S. Kowalski A. Choi L. Tack D. DeNieu M.. (2017). How well do you know your mutation? complex effects of genetic background on expressivity, complementation, and ordering of allelic effects. PLoS Genet. 13:e1007075. 10.1371/journal.pgen.1007075 29166655 PMC5718557

[B11] Clark A. G. (2004). The role of haplotypes in candidate gene studies. Genet. Epidemiol. 27, 321–333. 10.1002/gepi.20025 15368617

[B12] Datta A. Banerjee S. Hodges J. S. Gao L. (2019). Spatial disease mapping using directed acyclic graph auto-regressive (dagar) models. Bayesian Anal. 14, 1221–1244. 10.1214/19-BA1177 PMC8046356 33859772

[B13] de los Campos G. Vazquez A. I. Hsu S. Lello L. (2018). Complex-trait prediction in the era of big data. Trends Genet. 34, 746–754. 10.1016/j.tig.2018.07.004 30139641 PMC6150788

[B14] Duchen P. Leuenberger C. Szilágyi S. M. Harmon L. Eastman J. Schweizer M.. (2017). Inference of evolutionary jumps in large phylogenies using Lévy processes. Syst. Biol. 66, 950–963. 10.1093/sysbio/syx028 28204787 PMC5790141

[B15] Ewens W. J. (1972). The sampling theory of selectively neutral alleles. Theoret. Popul. Biol. 3, 87–112. 10.1016/0040-5809(72)90035-4 4667078

[B16] Ewens W. J. (2004). Mathematical Population Genetics 1, 2nd Edn. New York, NY: Springer-Verlag 10.1007/978-0-387-21822-9

[B17] Felsenstein J. (1988). Phylogenies and quantitative characters. Annu. Rev. Ecol. Syst. 19, 445–471. 10.1146/annurev.es.19.110188.002305

[B18] Gardiner C. (2009). Stochastic Methods. A Handbook for the Natural and Social Sciences, 4th Edn. Berlin; Heidelberg: Springer.

[B19] Gibson G. (2018). Population genetics and gwas: a primer. PLoS Biol. 16:e2005485. 10.1371/journal.pbio.2005485 29547618 PMC5877871

[B20] Gneiting T. Raftery A. E. (2007). Strictly proper scoring rules, prediction, and estimation. J. Am. Stat. Assoc. 102, 359–378. 10.1198/016214506000001437

[B21] Hadfield J. Nakagawa S. (2010). General quantitative genetic methods for comparative biology: phylogenies, taxonomies and multi-trait models for continuous and categorical characters. J. Evol. Biol. 23, 494–508. 10.1111/j.1420-9101.2009.01915.x 20070460

[B22] Hansen T. F. Martins E. P. (1996). Translating between microevolutionary process and macroevolutionary patterns: the correlation structure of interspecific data. Evolution 50, 1404–1417. 10.1111/j.1558-5646.1996.tb03914.x 28565714

[B23] Henderson C. R. (1976). A simple method for computing the inverse of a numerator relationship matrix used in prediction of breeding values. Biometrics 32, 69–83. 10.2307/2529339

[B24] Hickey J. Kinghorn B. Tier B. Clark S. A. van der Werf J. Gorjanc G. (2013). Genomic evaluations using similarity between haplotypes. J. Anim. Breed. Genet. 130, 259–269. 10.1111/jbg.12020 23855628

[B25] Hickey J. M. Chiurugwi T. Mackay I. Powell W. Eggen A. Kilian A.. (2017). Genomic prediction unifies animal and plant breeding programs to form platforms for biological discovery. Nat. Genet. 49:1297. 10.1038/ng.3920 28854179

[B26] Housworth E. A. Martins E. P. Lynch M. (2004). The phylogenetic mixed model. Am. Natural. 163, 84–96. 10.1086/380570 14767838

[B27] Huey R. B. Garland T. Jr. Turelli M. (2019). Revisiting a key innovation in evolutionary biology: Felsenstein's “phylogenies and the comparative method”. Am. Natural. 193, 755–772. 10.1086/703055 31094602

[B28] Ibanez-Escriche N. Simianer H. (2016). Animal breeding in the genomics era [Special issue]. Anim. Front. 6, 4–5. 10.2527/af.2016-0001

[B29] Johnsson M. Whalen A. Ros-Freixedes R. Gorjanc G. Chen C.-Y. Herring W. O. (2020). Genetics of recombination rate variation in the pig. bioRxiv. 10.1101/2020.03.17.995969

[B30] Kelleher J. Etheridge A. M. McVean G. (2016). Efficient coalescent simulation and genealogical analysis for large sample sizes. PLoS Comput. Biol. 12:e1004842. 10.1371/journal.pcbi.1004842 27145223 PMC4856371

[B31] Kelleher J. Wong Y. Wohns A. W. Fadil C. Albers P. K. McVean G. (2019). Inferring whole-genome histories in large population datasets. Nat. Genet. 51, 1330–1338. 10.1038/s41588-019-0483-y 31477934 PMC6726478

[B32] Koller D. Friedman N. (2009). Probabilistic Graphical Models: Principles and Techniques. Cambridge, MA: MIT Press.

[B33] Krainski E. T. Gómez-Rubio V. Bakka H. Lenzi A. Castro-Camilo D. Simpson D. (2018). Advanced Spatial Modeling with Stochastic Partial Differential Equations Using R and INLA. Boca Raton, FL: Chapman and Hall/CRC 10.1201/9780429031892

[B34] Lande R. (1976). Natural selection and random genetic drift in phenotypic evolution. Evolution 30, 314–334. 10.1111/j.1558-5646.1976.tb00911.x 28563044

[B35] Landis M. J. Schraiber J. G. Liang M. (2012). Phylogenetic analysis using lévy processes: finding jumps in the evolution of continuous traits. Syst. Biol. 62, 193–204. 10.1093/sysbio/sys086 23034385 PMC3566600

[B36] Leigh J. W. Bryant D. (2015). Popart: Full-feature software for haplotype network construction. Methods Ecol. Evol. 6, 1110–1116. 10.1111/2041-210X.12410

[B37] Lello L. Avery S. G. Tellier L. Vazquez A. I. de los Campos G. Hsu S. D. (2018). Accurate genomic prediction of human height. Genetics 210, 477–497. 10.1534/genetics.118.301267 30150289 PMC6216598

[B38] Lindgren F. Rue H. (2008). On the second-order random walk model for irregular locations. Scand. J. Stat. 35, 691–700. 10.1111/j.1467-9469.2008.00610.x

[B39] Liu F. Schmidt R. H. Reif J. C. Jiang Y. (2019). Selecting closely-linked snps based on local epistatic effects for haplotype construction improves power of association mapping. Genes Genomes Genet. 9, 4115–4126. 10.1534/g3.119.400451 31604824 PMC6893203

[B40] Lynch M. (1991). Methods for the analysis of comparative data in evolutionary biology. Evolution 45, 1065–1080. 10.1111/j.1558-5646.1991.tb04375.x 28564168

[B41] Maier R. M. Zhu Z. Lee S. H. Trzaskowski M. Ruderfer D. M. Stahl E. A.. (2018). Improving genetic prediction by leveraging genetic correlations among human diseases and traits. Nat. Commun. 9:989. 10.1038/s41467-017-02769-6 29515099 PMC5841449

[B42] Marchini J. (2019). Haplotype estimation and genotype imputation, in Handbook of Statistical Genomics, eds Balding D. Moltke I. Marioni J. (Oxford: John Wiley & Sons Ltd.), 87–114. 10.1002/9781119487845.ch3

[B43] Martins E. P. Hansen T. F. (1997). Phylogenies and the comparative method: a general approach to incorporating phylogenetic information into the analysis of interspecific data. *Am*. ist 149, 646–667. 10.1086/286013

[B44] McLaren W. Gil L. Hunt S. E. Riat H. S. Ritchie G. R. Thormann A.. (2016). The ensembl variant effect predictor. Genome Biol. 17:122. 10.1186/s13059-016-0974-4 27268795 PMC4893825

[B45] Meuwissen T. H. E. Hayes B. J. Goddard M. E. (2001). Prediction of total genetic value using genome-wide dense marker maps. Genetics 157, 1819–1829. Available online at: https://www.genetics.org/content/genetics/157/4/1819 11290733 10.1093/genetics/157.4.1819 PMC1461589

[B46] Misztal I. (2016). Inexpensive computation of the inverse of the genomic relationship matrix in populations with small effective population size. Genetics 202, 401–409. 10.1534/genetics.115.182089 26584903 PMC4788224

[B47] Morris A. P. Cardon L. R. (2019). Chapter 21: Genome-wide association studies, in Handbook of Statistical Genomics: Two Volume Set, 4th Edn. eds Balding D. Moltke I. Marioni J. (Oxford: John Wiley and Sons, Ltd), 597–550. 10.1002/9781119487845.ch21

[B48] Pagel M. (1999). Inferring the historical patterns of biological evolution. Nature 401:877. 10.1038/44766 10553904

[B49] Paradis E. (2014). Modern phylogenetic comparative methods and their application in evolutionary biology: concepts and practice, in Simulation of Phylogenetic Data, ed Garamszegi L. Z. (Berlin; Heidelberg: Springer), 335–350. 10.1007/978-3-662-43550-2_13

[B50] Puigbó P. Wolf Y. I. Koonin E. V. (2013). Seeing the tree of life behind the phylogenetic forest. BMC Biol. 11:46 10.1186/1741-7007-11-46 23587361 PMC3626908

[B51] Quaas R. (1988). Additive genetic model with groups and relationships. J. Dairy Sci. 71, 1338–1345.

[B52] R Core Team (2018). R: A Language and Environment for Statistical Computing. Vienna: R Foundation for Statistical Computing.

[B53] Rue H. Held L. (2005). Gaussian Markov Random Fields: Theory and Applications. Boca Raton, FL: Chapman and Hall/CRC 10.1201/9780203492024

[B54] Rue H. Martino S. Chopin N. (2009). Approximate Bayesian inference for latent Gaussian models by using integrated nested Laplace approximations. J. R. Stat. Soc. Ser. B 71, 319–392. 10.1111/j.1467-9868.2008.00700.x25257036

[B55] Rue H. Riebler A. Sørbye S. H. Illian J. B. Simpson D. P. Lindgren F. K. (2017). Bayesian computing with INLA: a review. Annu. Rev. Stat. Appl. 4, 395–421. 10.1146/annurev-statistics-060116-05404523481252

[B56] Schliep K. Potts A. A. Morrison D. A. Grimm G. W. (2017). Intertwining phylogenetic trees and networks. Methods Ecol. Evol. 8, 1212–1220. 10.1111/2041-210X.12760

[B57] Schraiber J. G. Landis M. J. (2015). Sensitivity of quantitative traits to mutational effects and number of loci. Theoret. Popul. Biol. 102, 85–93. 10.1016/j.tpb.2015.03.005 25840144 PMC4437879

[B58] Simons Y. B. Bullaughey K. Hudson R. R. Sella G. (2018). A population genetic interpretation of gwas findings for human quantitative traits. PLoS Biol. 16:e2002985. 10.1371/journal.pbio.2002985 29547617 PMC5871013

[B59] Simpson D. Rue H. Riebler A. Martins T. G. Sørbye S. H. (2017). Penalising model component complexity: a principled, practical approach to constructing priors. Stat. Sci. 32, 1–28. 10.1214/16-STS576

[B60] Sørbye S. H. Rue H. (2017). Penalised complexity priors for stationary autoregressive processes. J. Time Ser. Anal. 38, 923–935. 10.1111/jtsa.12242

[B61] Steyn Y. Lourenco D. A. L. Misztal I. (2019). Genomic predictions in purebreds with a multi-breed genomic relationship matrix. J. Anim. Sci. 97, 4418–4427. 10.1093/jas/skz258.099 31539424 PMC6827462

[B62] Suchard M. A. Lemey P. Baele G. Ayres D. L. Drummond A. J. Rambaut A. (2018). Bayesian phylogenetic and phylodynamic data integration using beast 1.10. Virus Evol. 4:vey016. 10.1093/ve/vey016 29942656 PMC6007674

[B63] Templeton A. R. Boerwinkle E. Sing C. F. (1987). A cladistic analysis of phenotypic associations with haplotypes inferred from restriction endonuclease mapping. I. basic theory and an analysis of alcohol dehydrogenase activity in Drosophila. Genetics 117, 343–351. 2822535 10.1093/genetics/117.2.343 PMC1203209

[B64] Thompson K. L. (2013). Using ancestral information to search for quantitative trait loci in genome-wide association studies (Ph.D. thesis). The Ohio State University. Columbus, OH, United States. 10.1186/1471-2105-14-200 23786262 PMC3706278

[B65] Uricchio L. H. (2019). Evolutionary perspectives on polygenic selection, missing heritability, and gwas. Hum. Genet. 139, 5–21. 10.1007/s00439-019-02040-6 31201529 PMC8059781

[B66] Uyeda J. C. Zenil-Ferguson R. Pennell M. W. (2018). Rethinking phylogenetic comparative methods. Syst. Biol. 67, 1091–1109. 10.1093/sysbio/syy031 29701838

[B67] Villumsen T. M. Janss L. Lund M. S. (2009). The importance of haplotype length and heritability using genomic selection in dairy cattle. J. Anim. Breed. Genet. 126, 3–13. 10.1111/j.1439-0388.2008.00747.x 19207924

[B68] Walsh B. Lynch M. (2018). Evolution and Selection of Quantitative Traits. Oxford: Oxford University Press 10.1093/oso/9780198830870.001.0001

[B69] Wojcik G. L. Graff M. Nishimura K. K. Tao R. Haessler J. Gignoux C. R.. (2019). Genetic analyses of diverse populations improves discovery for complex traits. Nature. 570, 514–518. 10.1038/s41586-019-1310-4 31217584 PMC6785182

[B70] Wu P. Hou L. Zhang Y. Zhang L. (2020). Phylogenetic tree inference: a top-down approach to track tumor evolution. Front. Genet. 10:1371. 10.3389/fgene.2019.01371 32117420 PMC7020887

